# NEIGHBORHOOD ENVIRONMENT AND DEPRESSIVE SYMPTOMS OF OLDER ADULTS IN URBAN AND RURAL CHINA : A MODEL OF SOCIAL CAPITAL

**DOI:** 10.1093/geroni/igad104.3493

**Published:** 2023-12-21

**Authors:** Yuekang Li

**Affiliations:** North China Electric Power University, Beijing, Beijing, China (People’s Republic)

## Abstract

Depression is a significant mental health issue among older adults worldwide. This study aimed to examine the mechanisms linking neighborhood environment to depressive symptoms among Chinese older adults and how other levels of social environment influence this relationship. Using data from the China Family Panel Studies, mediation and moderated mediation models were tested among 3,483 urban and 3,812 rural adults aged 60+ years. Results showed that community-level social capital (trust, reciprocity, belonging) partially mediated the association between built neighborhood environment and depressive symptoms in both urban and rural areas. The indirect pathway linking neighborhood environment to fewer depressive symptoms through community social capital was more evident when older adults reported less society social capital, suggesting a compensatory relationship between these two types of social capital. However, this relationship was significant only for urban older adults. The findings highlight the importance of distinguishing different levels of older adults’ social environment. Fostering community social capital through improving neighborhood walkability, safety, and amenities could benefit older adults’ mental health. Community development efforts may play a compensatory role for urban older adults lacking societal-level social resources.

